# Prevalence and associated factors of safe and improved infant and young children stool disposal in Ethiopia: evidence from demographic and health survey

**DOI:** 10.1186/s12889-019-7325-9

**Published:** 2019-07-22

**Authors:** Biniyam Sahiledengle

**Affiliations:** Department of Public Health, Madda Walabu University Goba Referral Hospital, Bale-Goba, Ethiopia

**Keywords:** Safe stool disposal, Improved disposal, Child stool, EDHS, Ethiopia

## Abstract

**Background:**

Infant and young children stools are often considered innocuous, and are not disposed of safely despite having a higher pathogen load than adult feces. In Ethiopia, sanitary management of young children’s stool is often overlooked and transmission of fecal-oral diseases is still a significant health burden. The study, therefore, describes the prevalence and associated factors of safe and improved child stool disposal.

**Methods:**

Data from the fourth round of the Ethiopian Health and Demographic Survey (EDHS) conducted in 2016 was used for this analysis. Descriptive statistics were computed. Bivariate and multivariable logistic regression analyses were performed to identify factors associated with safe and improved child stool disposal.

**Results:**

The prevalence of safe and improved child stool disposal in Ethiopia was 36.9% (95%CI: 33.4–40.5%) and 5.3% (95%CI: 4.3–6.5%) respectively. There was regional variation in the prevalence of safe and improved child stool disposal. The odds of safe stool disposal among households with richest wealth index had 4.54 (AOR: 4.54; 95%CI: 2.89–7.12), richer 3.64 (AOR: 3.64; 95%CI: 2.46–5.38), middle 3.26 (AOR: 2.26; 95%CI: 2.27–4.68), and poorer 1.93 (AOR: 1.93; 95%CI: 1.39–2.68) times higher odds of practicing safe child stool disposal than households with poorest wealth index. Similarly, households found in richest, richer, middle, and poorer wealth index had also (AOR: 20.23; 95%CI: 8.59–47.66), (AOR: 12.53; 95%CI: 5.59–28.10) (AOR: 4.91; 95%CI: 1.92–12.55), and (AOR: 4.50; 95%CI: 2.06–9.84) higher odds of practicing improved child stool disposal than households from poorest wealth index respectively. The odds of safe child stool disposal were higher among households whose children age between 6 and 11 months (AOR: 1.57; 95%CI: 1.17–2.09), 12–17 months (AOR: 1.39; 95%CI: 1.00–1.95), and 18–23 months (AOR: 1.43; 95%CI: 1.03–1.99) than households whose children age between 0 and 5 months. The odds of safe child stool disposal were 1.31 (AOR: 1.31; 95%CI: 1.00–1.72) and 1.44 (AOR: 1.44; 95%CI: 1.04–2.01) times higher among mothers whose age between 25 and 34 and greater than 34 years compared to mothers whose age between 15 and 24 years, respectively. In addition, children’s stools are more likely to be disposed of safely in urban households than in rural households (AOR: 3.12; 95%CI: 1.86–5.22). The present study also revealed households with access to improved sanitation facilities fail to use them for disposal of child stool (AOR: 0.99; 95% CI: 0.67–1.45).

**Conclusions:**

The prevalence of safe and improved child stool disposal in Ethiopia was found to be very low. Household socio-demographic and economic determinate were the key factors associated with child stool disposal. Appropriate strategic interventions to ensure safe and improved child stool disposal in Ethiopia is necessary. In addition, integrating child stool management into the existing sanitation interventions programs should be strongly recommended.

## Background

Access to adequate and equitable sanitation and hygiene for all, to end open defecation is still an issue and a cross-cutting problem throughout the globe [1–3]. The Millennium Development Goal (MDG) on sanitation coverage has not progressed as planned and remains a daunting challenge and unfinished agenda for the current era of Sustainable Development Goals (SDGs) [1]. And the SDG, particularly Goal 6 Target 6.2 holds promise to “Achieve access to adequate and equitable sanitation and hygiene for all, and end open defecation” by 2025 [3]. According to WHO/UNICEF, Joint Monitoring Programme (JMP) for Water Supply and Sanitation report globally, about 1 billion people practice open defecation, and an estimated 2.4 billion people lived without improved sanitation facilities [4]. In Sub-Saharan Africa, it is estimated that 229 million populations continue to engage in open defecation [5]. On top of this, in this sub-region of Africa as well as in many developing countries safe disposal of child stool is given less attention and remain a huge sanitation problem [6–10]. There is also a widespread belief that the stools of infants and young children are not harmful. As a result, the safe management of children’s stools has been perennially neglected due to this misconception [10, 11].

In fact, there is evidence that children’s stool could be riskier than adult feces, due to a higher prevalence of diarrhea and pathogens-such as hepatitis A, rotavirus, and *E.coli* [11]. Moreover, young children are frequently infected with enteric pathogens and their stools are actually an important source of infection [9]. And children whose stools were disposed of unsafely had higher odds of diarrhea prevalence [9]. A recent meta-analysis on children’s feces disposal practice also confirmed that unsafe child feces disposal practices increased the risk of diarrheal diseases by 23% [11]. In this regard, the safe disposal of children’s feces is decisive and essential as the safe disposal of adults’ feces [8, 9, 11–15].

In Ethiopia, like many Sub-Saharan Africa countries, poor sanitation is a major cause of fecal–oral diseases, including diarrhea [16–19]. In particular, children under the age of five years are the most affected as they are prone to water-borne diseases. In addition, unsafe disposal of children’s feces may be an important contaminant in household environments, posing a high risk of exposure to infants and young children [8, 13]. A study by Azage et al. also reported that the stool of more than six out of ten children under five in Ethiopia is disposed of unsafely [8]. In this regard, Ethiopia needs to walk a long road to achieve hygienic collection and disposal of young children’s feces [8, 13]. On one hand, only 6% of Ethiopian households use improved toilet facilities (16% in urban areas and 4% in rural areas) according to the recent EDHS 2016 report [1]. Even among households with improved toilets or latrines, almost half (49%) reported unsafe child feces disposal practice [13]. On the other hand, a very young child may not be able to use an improved toilet or sanitation facility because of their age and stage of physical development, even if their household has access to improved sanitation facility [13]. As a result, strengthening efforts to change the behavior of mothers and caregivers through programs and activities that aimed to filled knowledge and that encourage safe collection and disposal of child stool are crucial. On top of this, the prevalence of diarrhea increases after age 6 months, from 8% among children under age 6 months to 23% among those 6–11 months, and remains high (18%) at age 12–23 months, which is the time when children begin walking and are at increased risk of contamination from the environment [1].

The mini Ethiopian Demographic and Health Survey (EDHS) 2014 report showed open defection remains a significant problem in Ethiopia with a national rate of 34.1% (37.9% in rural and 8.7% in urban) [20]. In effect, the Ministry of Health of Ethiopia has implemented a number of initiatives long ago and currently being run, to increase sanitation and create awareness of the risks associated with open defecations [21, 22]. Moreover, Ethiopia’s launched a “National Hygiene and Sanitation Strategy: To Enable 100 percent Adoption of Improved Hygiene and Sanitation”, which focus on eliminating the practice of open defecation [22–24]. Despite the efforts to date in Ethiopia, it is unclear how progress has affected the practice of different segments of sub-populations, in particular, young children’s stool disposal practice. From the available evidence, the practice of child feces disposal of mothers has only been documented in a few pieces of literature [8]. Even the formerly conducted study did not assess the prevalence and associated factors of improved child feces disposal. To the best of the author’s knowledge, this is the first study in Ethiopia that uses a large-scale population-based representative dataset to assess the association between socio-demographic, economic and environmental variables and improved child stool disposal. The study, therefore, aims to describe the prevalence and associated factors of safe and improved child stool disposal in Ethiopia.

## Methods

### Study design, setting, and data

The study was conducted following the methodology presented by the Central Statistical Agency (CSA) and ICF [1]. And the recent nationally representative population-based Ethiopian Demographic and Health Survey (EDHS-4) data conducted in 2016 was used in this analysis. The sample is representative at a national, residence (i.e., urban/rural), and regional level. The samples were selected using a two-stage stratified cluster sampling technique with regions and residence as strata. Initially, all nine regions were stratified into urban and rural clusters. From 645 enumeration areas, 202 urban and 443 rural clusters were considered. In the second stage of selection, a fixed number of 28 households per cluster were selected from the newly updated listing of households. Altogether, 16,650 households and 15,683 women aged 15–49 years were interviewed in the survey. The response rates were 98 and 95%, respectively.

The study included all youngest child under age two living with the mother from each household and mothers were asked about the disposal practice of the last passed stool with respect to the youngest child.

### Study variables

The outcome variables for this study were the disposal practice of children’s stool, “safe/unsafe” and “improved/unimproved”. Mothers of children were asked, “The last time passed stools, what was done to dispose of the stools”? The response included: ‘child used the toilet or latrine,’ ‘put/rinsed into toilet or latrine,’ ‘put/rinsed into drain/ditch,’ ‘thrown into the garbage,’ ‘buried,’ ‘left in the open,’ and ‘other.’ The outcome variables were constructed based on the WHO definition, response categories such as ‘child used toilet or latrine’ and ‘put/rinsed into toilet or latrine’ were combined and coded as ‘safe disposal of child stool (coded as ‘1’) [25]. And the others were coded as ‘unsafe disposal of child stool (coded as ‘0’)’. Similarly, improved child’s stool disposal was coded as ‘1’ when a child’s stools were put or rinsed into an “improved” toilet/latrine or child used toilet/latrine and ‘0’ otherwise.

Explanatory variables such as socioeconomic, demographic and environmental factors from the EDHS-4 dataset were extracted for further analysis. The variables include; household’s wealth (poorest, poorer, middle, richer, richest), sex of children, age of the child (0–5 months, 6–11 months, 12–17 months, 18–23 months), mother’s age (15–24, 25–34, > 34), mother educational level (no education, primary, secondary, higher), region, place of residence (urban, rural), religion, mother’s exposure to media, toilet facility (improved, unimproved), sources of drinking water (improve, unimproved) and presence of diarrhea in the last two weeks (yes, no). The variable on media exposure includes exposure to newspaper, television, and radio. The mothers who were not exposed to each media were coded as “no” and those who have frequent exposure were coded as “yes”. In addition, the toilet facility and source of drinking water were categorized into ‘improved’ and ‘unimproved’ following the WHO/UNICEF definition [15].

### Statistical analysis

The analysis was carried out in SPSS version 20 software. Appropriate sampling weights were used in the estimations for the adjustment of cluster sampling design. A complex sample binary logistic regression model was employed to assess the association between the explanatory variables and the outcome variables. Chi-square test was also used to describe child stool disposal by the explanatory variables. Bivariate and multivariable logistic regression analyses were applied with α = 0.05 as a cut-off point for all statistically significant tests.

## Results

### Household characteristics and child stool disposal

Data about safe and improved stool disposal characteristics were analyzed using 4,145 youngest children under age two living with the mother from the 2016 EDHS. Table 1 shows the percentage of youngest children’s stools disposal. Overall, stools of 36.9% (95%CI: 33.4–40.5%) of children in Ethiopia were only disposed of safely. And only 5.3% (95%CI: 4.3–6.5%) of children stools were disposed by means of an improved sanitation facility.

**Table 1:** Weighted prevalence of youngest children’s stool disposal in Ethiopia, EDHS 2016 (*n* = 4145)

Child feces disposal practices	Weighted frequency	Weighted percent	95% CI
Used toilet/latrine	30	0.7	0.4–1.2
Put/rinsed in toilet/latrine	1499	36.2	32.8–39.7
Put/rinsed into drain or ditch	155	3.7	2.8–5.0
Throw into garbage	758	18.3	16.3–20.5
Buried	117	2.8	2.1–3.8
Left in the open/not disposed of	1055	25.5	22.1–29.1
Other	529	12.8	10.9–14.9
Overall children’s stool disposal practice			
Safe ♣	1530	36.9	33.4–40.5
Unsafe	2615	63.1	59.5–66.6
Overall children’s improved stool disposal			
Improved †	216	5.3	4.3–6.5
Unimproved	3929	94.7	93.5–95.7

♣ Safe disposal of children’s stools: the child’s last feces were put in or rinsed into a toilet or latrine, or the child used a toilet or latrine

†When a child’s feces is put or rinsed into an “improved” toilet or latrine, this is termed “improved child feces disposal”

Tables 2 and 3 show the child’s stool disposal by the socio-demographic and socio-economic characteristics. More than half (56.2%) of the households used an improved source of drinking water and only (10.1%) of the households used improved toilet facility. Regarding diarrhea prevalence, 16.2% of young children experienced diarrhea in the last two weeks preceding the survey.

**Table 2:** Child’s stool disposal by selected socio-demographic and socio-economic characteristics in Ethiopia, EDHS 2016 (*N* = 4145)

Background characteristics	Child’s stool disposal practice		Total	Percent	X^2^ (df), *P*-value
	Safe	Unsafe			
Region					
Tigray	88	216	304	7.3	291.9 (10), p-value = 0.000
Affar	10	30	40	1.0	
Amhara	261	499	760	18.3	
Oromiya	532	1316	1848	44.6	
Somali	42	129	171	4.1	
Benishangul	22	22	44	1.1	
SNNP	508	328	836	20.2	
Gambela	3	6	9	0.2	
Harari	4	6	10	0.2	
Addis Ababa	50	55	105	2.5	
Dire Dawa	10	8	18	0.4	
Place of residence					
Urban	297	201	498	12.0	125.5 (1), *p*-value = 0.000
Rural	1233	2414	3647	88.0	
Mother educational level					
No education	801	1699	2500	60.3	91.3 (3), *p*-value = 0.000
Primary	535	744	1279	30.9	
Secondary	120	134	254	6.1	
Higher	74	38	112	2.7	
Religion (*n* = 4144)					
Orthodox	502	902	1407	34.0	168.4 (5), *p*-value = 0.000
Catholic	12	29	41	1.0	
Protestant	469	389	858	20.7	
Muslin	515	1211	1726	41.7	
Traditional	11	59	70	1.7	
Other	16	26	42	1.0	
Household wealth index (*n* = 4144)					
Poorest	171	740	911	22.0	247.3(4), *p*-value = 0.000
Poorer	274	629	903	21.8	
Middle	381	500	881	21.3	
Richer	345	398	743	17.9	
Richest	358	348	706	17.0	
Listening to radio					
Yes	529	601	1130	27.3	65.4(1), *p*-value = 0.000
No	1001	2014	3015	72.7	
Watching television					
Yes	417	345	763	18.4	127.4(1), *p*-value = 0.000
No	1112	2270	3382	81.6	
Reading the newspaper or magazine					
Yes	160	125	285	6.9	48.5(1), *p*-value = 0.000
No	1370	2490	3860	93.1	
Sex of child (n = 4144)					
Male	697	1283	1980	47.8	4.6(1), *p*-value = 0.039
Female	832	1332	2164	52.2	
Diarrhea in the last two weeks (*n* = 4129)					
Yes	305	365	670	16.2	25.0(1), *p*-value = 0.000
No	1222	2237	3459	83.8	
Toilet facility					
Improved^a^	216	203	419	10.1	42.8(1), *p*-value = 0.000
Unimproved	1314	2412	3726	89.9	
Source of drinking water					
Improved^b^	966	1364	2330	56.2	47.5(1), *p*-value = 0.000
Unimproved	563	1251	1815	43.8	
Age of the child (n = 4144)					
0–5 months	356	831	1187	28.6	36.6(3), *p*-value = 0.000
6–11 months	438	621	1059	25.6	
12–17 months	412	672	1084	26.2	
18–23 months	323	491	814	19.6	
Mother’s age					
15–24	373	842	1215	29.3	28.8(2), *p*-value = 0.000
25–34	839	1267	2106	50.8	
> 34	318	506	824	19.9	

^a^Facilities that would be considered improved if they were not shared by two or more households

^b^Include piped water, public taps, standpipes, tube wells, boreholes, protected dug wells and springs, rainwater and bottled water

**Table 3:** Improved child’s stool disposal by selected socio-demographic and socio-economic characteristics in Ethiopia, EDHS 2016 (N = 4145)

Background characteristics	Improved child’s feces disposal practice		Total	Percent	X^2^ (df), *p*-value
	Improved	Unimproved			
Region					
Tigray	33	271	304	7.3	375.37(10), p-value = 0.000
Affar	2	37	39	0.9	
Amhara	14	747	761	18.4	
Oromiya	43	1805	1848	44.6	
Somali	29	142	171	4.1	
Benishangul	1	43	44	1.1	
SNNP	50	786	836	20.2	
Gambela	1	9	10	0.2	
Harari	2	8	10	0.2	
Addis Ababa	40	65	105	2.5	
Dire Dawa	6	11	17	0.4	
Place of residence					
Urban	133	365	498	12.0	512.35(1), *p*-value = 0.000
Rural	83	3559	3647	88.0	
Mother educational level					
No education	62	2437	2500	60.3	253.09(3), *p*-value = 0.000
Primary	78	1201	1279	30.9	
Secondary	48	206	254	6.1	
Higher	32	80	112	2.7	
Religion					
Orthodox	92	1315	1407	33.9	11.99(5), *p*-value = 0.035
Catholic	0	41	41	1.0	
Protestant	45	814	859	20.7	
Muslin	81	1645	1726	41.6	
Traditional	0	70	70	1.7	
Other	3	39	42	1.0	
Household wealth index (n = 4144)					
Poorest	7	968	975	23.5	489.97(4), *p*-value = 0.000
Poorer	14	891	905	21.8	
Middle	15	852	867	20.9	
Richer	36	718	754	18.2	
Richest	148	495	643	15.5	
Listening to radio					
Yes	105	1024	1129	27.2	48.41(1), *p*-value = 0.000
No	116	2900	3016	72.8	
Watching television					
Yes	138	625	763	18.4	301.404(1), *p*-value 0.000
No	83	3299	3382	81.6	
Reading the newspaper or magazine					
Yes	53	232	285	6.9	107.49(1), *p*-value = 0.000
No	167	3692	3859	93.1	
Sex of child					
Male	103	1877	1980	47.8	0.13(1), *p*-value = 0.722
Female	118	2047	2165	52.2	
Diarrhea in the last two weeks (n = 4129)					
Yes	39	631	670	16.2	0.35(1), *p*-value = 0.556
No	182	3277	3459	83.8	
Toilet facility					
Improved*	221	198	419	10.1	2075.95(1), *p*-value = 0.000
Unimproved	0	3726	3726	89.9	
Source of drinking water					
Improved	187	2143	2330	56.2	76.51(1), *p*-value = 0.000
Unimproved	34	1781	1815	43.8	
Age of the child					
0–5 months	46	1141	1187	28.6	7.13(3), *p*-value = 0.000
6–11 months	64	995	1059	25.5	
12–17 months	62	1023	1085	26.2	
18–23 months	49	765	814	19.6	
Mother’s age					
15–24	60	1156	1216	29.3	2.32(2), *p*-value = 0.314
25–34	123	1982	2105	50.8	
> 34	38	786	824	19.9	

### Factors associated with safe child stool disposal

Table 4 shows the result of the bivariate and multivariable logistic regression analyses of factors associated with children’s stool disposal. In bivariate logistic regression analysis region, place of residence, mother educational level, religion, household wealth index, listening to radio, watching television, reading the newspaper or magazine, diarrhea in the last two weeks, age of the child, mother’s age, toilet facility and source of drinking water were factors associated with safe child stool disposal.

**Table 4:** Factors associated with safe children’s stool disposal in Ethiopia, EDHS 2016

Background characteristics	Child’s stool disposal practice		COR (95% CI)	AOR (95% CI)
	Safe	Unsafe		
Region				
Tigray	88	216	0.32(0.17–0.60)*	0.40 (0.17–0.90)**
Affar	10	30	0.26(0.13–0.52)*	0.65 (0.29–1.46)
Amhara	261	499	0.42(0.22–0.77)*	0.59(0.26–1.30)
Oromiya	532	1316	0.32(0.18–0.57)*	0.45(0.22–0.92)**
Somali	42	129	0.26(0.14–0.47)*	0.67(0.33–1.36)
Benishangul	22	22	0.77(0.41–1.43)	1.45(0.67–3.15)
SNNP	508	328	1.24(0.71–2.17)	1.65(0.74–3.69)
Gambela	3	6	0.40(0.20–0.79)*	0.48(0.21–1.09)
Harari	4	6	0.57(0.30–1.08)	0.51(0.23–1.13)
Addis Ababa	50	55	0.72(0.39–1.32)	0.17(0.07–0.40)**
Dire Dawa	10	8	1	1
Place of residence				
Urban	297	201	2.88(1.95–4.26)*	3.12(1.86–5.22)**
Rural	1233	2414	1	1
Mother educational level				
No education	801	1699	1	1
Primary	535	744	1.52(1.21–1.91)*	1.12(0.86–1.46)
Secondary	120	134	1.89(1.24–2.87)*	0.78(0.50–1.21)
Higher	74	38	4.16(2.27–7.63)*	0.93(0.48–1.79)
Religion (n = 4144)				
Orthodox	502	902	1	1
Catholic	12	29	0.75(0.24–2.35)	0.69(0.22–2.11)
Protestant	469	389	2.14(1.50–3.06)*	1.36(0.86–2.16)
Muslin	515	1211	0.75(0.54–1.05)	1.06(0.69–1.63)
Traditional	11	59	0.34(0.09–1.33)	1.02(0.49–2.11)
Other	16	26	1.14(0.37–3.47)	1.07(0.38–2.99)
Household wealth index (n = 4144)				
Poorest	171	740	1	1
Poorer	274	629	1.89(1.33–2.67)*	1.93(1.39–2.68)**
Middle	381	500	3.30(2.32–4.70)*	3.26(2.27–4.68)**
Richer	345	398	3.76(2.54–5.55)*	3.64(2.46–5.38)**
Richest	358	348	4.46(2.96–6.71)*	4.54(2.89–7.12)**
Listening to radio(n = 4144)				
Yes	529	601	1.77(1.40–2.23)*	1.18(0.87–1.60)
No	1001	2014	1	1
Watching television				
Yes	417	345	2.46(1.84–3.31)*	1.45(0.99–2.12)
No	1112	2270	1	1
Reading the newspaper or magazine				
Yes	160	125	2.31(1.56–3.42)*	1.21(0.77–1.89)
No	1370	2490	1	1
Sex of child (n = 4144)				
Male	697	1283	1	
Female	832	1332	1.14(0.94–1.40)	
Diarrhea in the last two weeks (n = 4129)				
Yes	305	365	1.52(1.17–1.97)*	1.27(0.97–1.68)
No	1222	2237	1	1
Toilet facility				
Improved	216	203	1.95(1.42–2.67)*	0.99(0.66–1.47)
Unimproved	1314	2412	1	1
Source of drinking water				
Improved	966	1364	1.57(1.19–2.07)*	1.04(0.80–1.36)
Unimproved	563	1251	1	1
Age of the child (n = 4144)				
0–5 months	356	831	1	1
6–11 months	438	621	1.64(1.28–2.11)*	1.57(1.17–2.09)**
12–17 months	412	672	1.43(1.05–1.94)*	1.39(1.00–1.95)**
18–23 months	323	491	1.53(1.16–2.03)*	1.43(1.03–1.99)**
Mother’s age				
15–24	373	842	1	1
25–34	839	1267	1.49(1.19–1.87)*	1.31(1.00–1.72)**
> 34	318	506	1.41(1.06–1.88)*	1.44(1.04–2.01)**

*CI* = Confidence Interval, *COR* = Crude Odds Ratio, *AOR* = Adjusted Odds Ratio, *Significant association (*P* < 0.05) crude, ** Significant association (*p* < 0.05) adjusted

In multivariable logistic regression analysis, the odds of disposing of stools safely were 60% lower (AOR: 0.40; 95%CI: 0.17–0.90), 55% lower (AOR: 0.45; 95%CI: 0.22–0.92) and 83% lower (AOR: 0.17; 95%CI: 0.07–0.40) among households in Tigray, Oromiya and Addis Ababa than Dire Dawa, respectively. Safe disposal of children’s stools was statistically associated with the household wealth index. The odds of safe stools disposal among households with poorer, middle, richer and richest wealth index had 1.93, 3.26, 3.64 and 4.54 times higher odds to practice safe child stool disposal than households with poorest wealth index (AOR:1.93; 95%CI: 1.39–2.68), (AOR: 3.26; 95%CI: 2.27–4.68), (AOR: 3.64; 95%CI: 2.46–5.38) and (AOR: 4.54; 95%: 2.89–7.12), respectively. Another variable that was statistically associated with safe disposal of stool was the age of the child and mother. The odds of safe child stool disposal were 1.57 times higher among households whose children age between 6 and 11 months (AOR: 1.57; 95%CI: 1.17–2.09), 1.39 times higher among households whose children age between 12 and 17 months (AOR: 1.39; 95%CI: 1.00–1.95), and 1.43 times higher among households whose children age between 18 and 23 months (AOR: 1.43; 95%CI: 1.03–1.99) compared to households whose children age between 0 and 5 months. Similarly, the odds of safe child stool disposal were 1.31 times higher among mothers whose age between 25 and 34 years old (AOR: 1.31; 95%CI: 1.00–1.72) and 1.44 times higher among mothers whose age greater than 34 years old compared to mothers whose age group were between 15 and 24 years old (AOR: 1.44; 95%CI: 1.04–2.01). In this study, children’s stools are more likely to be disposed of safely in urban households than in rural households (AOR: 3.12; 95%CI: 1.86–5.22). On the other hand, households with access to improved sanitation facilities fail to use them for disposal of child stool (AOR: 0.99; 95% CI: 0.67–1.45).

### Factors associated with improved child stool disposal

Table 5 presented the result of the bivariate and multivariable logistic regression analyses assessing the factors associated with improved children’s stool disposal. In bivariate logistic regression analysis region, place of residence, mother educational level, household wealth index, listening to radio, watching television, reading the newspaper or magazine, and source of drinking water were factors associated with improved child stool disposal. In multivariable logistic regression analysis, the odds of improved child stool disposal were 71, 75, 95, and 91% lower among households in Tigray (AOR: 0.29; 95%CI: 0.15–0.55), Affar (AOR: 0.25; 95%CI: 0.13–0.47), Amhara (AOR: 0.05; 95%: 0.02–0.19) and Oromiya (AOR: 0.09; 95%CI: 0.04–0.22) than Dire Dawa, respectively. Similarly, the odds of improved child stool disposal were 91, 73, 84, 63 and 74% lower among households in Benishangul (AOR: 0.09; 95%CI: 0.04–0.22), SNNP (AOR: 0.27; 95%CI: 0.12–0.59), Gambela (AOR: 0.16; 95%CI: 0.07–0.36), Harari (AOR: 0.37; 95%CI: 0.19–0.72), and Addis Ababa (AOR: 0.26; 95%CI: 0.13–0.51) than Dire Dawa, respectively. On the other than, households in the Somali region were 2.61 times (AOR: 2.61; 95%: 1.06–6.42) higher odds of improved child stool disposal compared to Dire Dawa. In the present study improved child stool disposal were associated with the household wealth index. The odds of improved child stool disposal among households with poorer, middle, richer and richest wealth index were 4.50, 4.91, 12.53 and 20.23 times higher compared to households with poorest wealth index (AOR: 4.50; 95%CI: 2.06–9.84), (AOR: 4.91; 95%CI: 1.92–12.55), (AOR: 12.53; 95%CI: 5.59–28.10) and (AOR: 20.23; 95%CI: 8.59–47.66), respectively. Mother’s exposure to television also another factor associated with improved child stool disposal. The odds of improved child stool disposal was 2.23 times higher (AOR: 2.23; 95%CI: 1.19–4.15) among mother who was watching television than those who were not at all.

**Table 5:** Factors associated with improved child’s stool disposal in Ethiopia, EDHS 2016

Background characteristics	Improved child’s feces disposal practice		COR (95% CI)	AOR (95% CI)
	Improved	Unimproved		
Region				
Tigray	33	271	0.22(0.13–0.38)*	0.29(0.15–0.55)**
Affar	2	37	0.11(0.05–0.25)*	0.25(0.13–0.47)**
Amhara	14	747	0.03(0.01–0.11)*	0.05(0.02–0.19)**
Oromiya	43	1805	0.04(0.02–0.09)*	0.09(0.04–0.22)**
Somali	29	142	0.36(0.19–0.65)*	2.61(1.06–6.42)**
Benishangul	1	43	0.04(0.02–0.10)*	0.09(0.04–0.22)**
SNNP	50	786	0.11(0.06–0.21)*	0.27(0.12–0.59)**
Gambela	1	9	0.17(0.09–0.35)*	0.16(0.07–0.36)**
Harari	2	8	0.45(0.24–0.83)*	0.37(0.19–0.72)**
Addis Ababa	40	65	1.08(0.61–1.92)	0.26(0.13–0.51)**
Dire Dawa	6	11	1	1
Place of residence				
Urban	133	365	14.77(9.29–23.49)*	1.859(0.90–3.84)
Rural	83	3559	1	1
Mother educational level				
No education	62	2437	1	1
Primary	78	1201	2.52(1.64–3.89)*	1.40(0.85–2.31)
Secondary	48	206	9.14(5.03–16.61)*	1.90(0.93–3.89)
Higher	32	80	15.27(8.20–28.45)*	1.62(0.73–3.62)
Household wealth index (n = 4144)				
Poorest	7	968	1	1
Poorer	14	891	2.12(1.02–4.42)*	4.50(2.06–9.84)**
Middle	15	852	2.33(0.88–6.19)*	4.91(1.92–12.55)**
Richer	36	718	6.65(3.10–14.27)*	12.53(5.59–28.10)**
Richest	148	495	39.43(20.22–76.88)*	20.23(8.59–47.66)**
Listening to radio				
Yes	105	1024	2.58(1.76–3.78)*	0.92(0.55–1.55)
No	116	2900	1	1
Watching television				
Yes	138	625	8.73(5.88–12.94)*	2.23(1.19–4.15)**
No	83	3299	1	1
Reading the newspaper or magazine				
Yes	53	232	5.08(3.30–7.81)*	0.99(0.51–1.91)
No	167	3692	1	1
Diarrhea in the last two weeks (n = 4129)				
Yes	39	631	1.11(0.68–1.82)	
No	182	3277	1	
Source of drinking water				
Improved	187	2143	4.58(2.78–7.54)*	1.55(0.91–2.66)
Unimproved	34	1781	1	1
Age of the child				
0–5 months	46	1141	1	
6–11 months	64	995	1.60(0.96–2.68)	
12–17 months	62	1023	1.52(0.96–2.39)	
18–23 months	49	765	1.59(0.92–2.74)	
Mother’s age				
15–24	60	1156	1	
25–34	123	1982	1.21(0.83–1.77)	
> 34	38	786	0.94(0.53–1.66)	

*CI* = Confidence Interval, *COR* = Crude Odds Ratio, *AOR* = Adjusted Odds Ratio, *Significant association (*P* < 0.05) crude, ** Significant association (*p* < 0.05) adjusted

## Discussion

This study reported the safe and improved child stool disposal practices of 4145 children under age two living with the mother in Ethiopia, together with the factors associated with these practices. Overall, the stool of 36.9 and 5.3% of children below two years of age was disposed of safely and with improved sanitation, respectively. Variables such as region, place of residence, household wealth index, the age of the child and age of the mother were the main factors associated with child stool disposal.

The prevalence of safe child stool disposal practice found in this study is almost similar to the prevalence reported by Azage et al., 33.68% [8] and other low-income settings, such as Madagascar [26] and Nepal [27]. Additionally, studies conducted in India and Bangladeshi also reported a similar low prevalence of safe child stool disposal [28–30]. The finding implies the majority of cases children’s stool was disposed of unsafely, which may possibly put a child at risk of infection through multiple pathways. And, when there is improper child’s stool disposal in the community, both adults and children are at risk of enteric infection and not just the children alone. There are also evidence regarding the association between unsafe excreta disposal and a high burden of diarrhea, soil-transmitted helminth infections, trachoma and other enteric diseases [12, 25]. In connection, a study conducted by Bawankule et al. reported children whose stools were disposed of unsafely were more likely to suffer from diarrhea than children whose stools were disposed of safely [9].

However, the present study did not detect such association, safe child stool disposal and decreased odds of diarrheal prevalence. Likewise, a study by Islam et al. also reported unsafe child feces disposal was not significantly associated with presences of diarrhea among children under age three [29]. The absence of such an association might be explained in a number of ways. The first reason might be due to the age category of children. This age category of children (age < 2 years) may not be able to use a toilet facility because of their age and stage of physical development. In addition, children under age 6 months and those 6–11 months were not beginning walking and less likely to exposed to a contaminated environment. Although the prevalence of diarrhea may not only depend on unsafe stool disposal but also psychosocial factors (feeding practice and nurturing), mother personal hygiene, and environmental sanitation. To overcome, such phenomenon improving access to sanitation facilities alone is not enough, however context-specific behavior change strategies equally important. Countries like Ethiopia, where the burden of childhood diarrhea is prevalent should explore opportunities to integrate child stool management into existing sanitation intervention programs that target mothers and caregivers of young children. Sanitation strategies such as educating mothers or caregivers on safe disposal of children’s stools along with building sanitation facilities are also essential in curbing the high prevalence of unsafe child stool disposal. Furthermore, the promotions of behavior change strategies to prevail over barriers to disposal of child stool and water used for child bathing after defecation should be considered [25].

In this study, the most common type of unsafe child stool disposal method was left child feces in the open or not disposed of (25.5%). Meaning a significant number of children stools were disposed of unsafely in open field, and if feces are left uncontained, diseases may spread by direct contact or animal contact [1, 25, 31]. Systematic studies also plainly indicated that diarrheal diseases were highly prevalent in areas where poor hygiene and lack of sanitation is widespread [11, 32]. In connection, literature documented that the practice of unsafe child stool disposal can cause environmental contamination by fecal pathogens that can cause enteric diseases among young children’s [10, 29, 30, 33, 34].

In this study, the odds of practicing safe disposal of child stool were increased with the increased level of household wealth index. Households from a higher wealth quintile were more likely to practice safe disposal of child stool than those households from the poorest wealth quintile. This finding is consistent with the studies from Ethiopia [8], India [9], South Africa [35] and Burkina Faso [36].

Place of residence was another factor that significantly associated with safe child stool disposal. Children’s stools are more likely to be disposed of safely in urban households than in rural households. Similar higher safe child stool disposal practice among urban residents was reported from a similar study from Ethiopia [8], and Kenya [37].

Ages of the child and mother’s age were the other factors that positively associated safe child stool disposal. This finding is consistent with the finding of a similar study conducted in Ethiopia [8] and Bangladesh [30, 31]. This could be explained by a shift in safe disposal practices seen as children grow; children are increasingly likely to use a toilet/latrine themselves, rather than have their feces put or rinsed into one [13]. And the old age mothers and caregivers may be more conscious and observant about disposing of child feces safely and are more likely to understand the causes of childhood illness.

In multivariable logistic regression analysis, the presence of an improved sanitation facility was not associated with safe child stool disposal. The comparable finding was reported from rural Bangladesh [30]. Rand et al. also reported, in 15 out of 26 locations more than 50% of households reported that the feces of their youngest child under three years were disposed of unsafely; even the percentage of feces ending up in improved sanitation facilities is much lower [14]. These findings suggested that even those with access to improved sanitation facilities often fail to use them for disposal of child feces [25, 31]. Meaning, people who are having improved toilets at their house are disposing of the child stool in a risky way.

In fact, access to sanitation facilities is a pre-requisite to ending open defecation as well as unsafe child stool disposal, but it is not always a sufficient condition to overcome unsafe child stool disposal [25, 38, 39]. A study by Phaswana-Mafuya et al. identified improvement and presence of physical sanitation infrastructure alone is not sufficient to ensure safe hygienic practices [35]. In overcome such situation, robust sanitation promotion and strong behavior change program that targeted on the determinants of behaviors is important.

The prevalence of improved child stool disposal found in this study (5.3%) is almost close to the prevalence reported in the last EDHS-3 (2011) 3.0% [13]. In fact, according to the most recent EDHS-4 report overall 6% of Ethiopian households use improved toilet facilities (16% in urban areas and 4% in rural areas) [1]. Subsequently, improved child stool disposal is only possible where there is access to improved sanitation facilities [13]. According to the recent WHO sanitation and health guideline, disposal of child feces in a toilet connected to a safe sanitation chain is the only safe method where solid waste management systems for children’s absorbent underclothes (nappies) disposal are not safe [25]. The association between place of residence and improved disposal of child feces in this study is not surprising since there is a significant variation in improved sanitation coverage among urban and rural residents in Ethiopia. In the present study, the household wealth index was a strong predictive factor for having improved child stool disposal. The finding is in line with other related studies [35, 40, 41].

This study has several limitations. First, it has all the disadvantages of any cross-sectional study; the temporal relationship between the outcome and independent variables could not be established. Second, mothers’ knowledge and perception towards safe and improved disposal of child feces were not assessed in this study. Moreover, the study may be susceptible to social desirability and recall bias, as the data dealt with reported practices rather than direct observation. The other limitation of this study was lack of exhaustiveness to include all the relevant variables, such as child stool collection practice that may influence the practice of safe and improved disposal of child stool. Furthermore, some of the regions had a small sample size, which questions the accuracy of prevalence estimates per region, so that it should be interpreted with caution.

## Conclusions

The prevalence of safe and improved child stool disposal in Ethiopia was found to be very low and a common sanitation problem. Children’s stools are more likely to be disposed of safely in urban households than in rural households. There is also regional variation in the prevalence of safe and improved child stool disposal in Ethiopia. A household with higher wealth index was one of the key factors associated with safe and improved child stool disposal. Child and maternal age were other factors associated with safe child stool disposal. Appropriate strategic interventions to ensure safe and improved child stool disposal in Ethiopia is necessary. There is still no strong effective strategy for reducing the unsafe disposal of child feces in Ethiopia, as a result, it is very important to explore possible ways to integrate and incorporating child sanitation into existing CLTS and other national hygiene and sanitation strategies to enable adoption of safe and improved sanitation at the community level. In addition, building toilets and having improved sanitation facilities is not enough in curbing the high prevalence of unsafe disposal of children’s stools in Ethiopia. Consequently, an effective strategy such as awareness creation and educating mothers and caregivers on the safe disposal of children’s stools is crucial.

## Data Availability

The dataset was demanded and retrieved from the DHS website https://dhsprogram.com after formal online registration and submission of the project title and detail project description.

## Abbreviations

AOR: Adjusted odds ratio
CI: Confidence interval
CLTS: Community-Led Total Sanitation
COR: Crude odds ratio
DHS: Health and demographic surveys
EDHS: Ethiopian Health and demographic surveys
SDGs: Sustainable Development Goals
SPSS: Statistical Package for Social Sciences
VIF: Variance inflation factor
WHO: World Health Organization
